# Two Cases of Heerfordt's Syndrome: A Rare Manifestation of Sarcoidosis

**DOI:** 10.1155/2016/3642735

**Published:** 2016-01-17

**Authors:** Keishi Fujiwara, Yasushi Furuta, Satoshi Fukuda

**Affiliations:** ^1^Department of Otolaryngology, Head and Neck Surgery, Graduate School of Medicine, Hokkaido University, N15W7, Kita-ku, Sapporo 0608638, Japan; ^2^Department of Otolaryngology, Head and Neck Surgery, Teine-Keijinkai Hospital, 12-1-40 Maeda 1-Jo, Teine-Ku, Sapporo 0068555, Japan

## Abstract

Heerfordt's syndrome is a rare manifestation of sarcoidosis characterized by the presence of facial nerve palsy, parotid gland enlargement, anterior uveitis, and low grade fever. Two cases of Heerfordt's syndrome and a literature review are presented.* Case  1*. A 53-year-old man presented with swelling of his right eyelid, right facial nerve palsy, and swelling of his right parotid gland. A biopsy specimen from the swollen eyelid indicated sarcoidosis and he was diagnosed with incomplete Heerfordt's syndrome based on the absence of uveitis. His symptoms were improved by corticosteroid therapy.* Case  2*. A 55-year-old woman presented with left facial nerve palsy, bilateral hearing loss, and swelling of her bilateral parotid glands. She had been previously diagnosed with uveitis and bilateral hilar lymphadenopathy. Although no histological confirmation was performed, she was diagnosed with complete Heerfordt's syndrome on the basis of her clinical symptoms. Swelling of the bilateral parotid glands and left facial nerve palsy were improved immediately by corticosteroid therapy. Sarcoidosis is a relatively uncommon disease for the otolaryngologist. However, the otolaryngologist may encounter Heerfordt's syndrome as this syndrome presents with facial nerve palsy and swelling of the parotid gland. Therefore, we otolaryngologists should diagnose and treat Heerfordt's syndrome appropriately in cooperation with pneumologists and ophthalmologists.

## 1. Introduction

Sarcoidosis is a systemic granulomatous disease of unknown etiology. Although it usually affects the lung, any organ may be involved. Heerfordt's syndrome is a rare manifestation of sarcoidosis characterized by the presence of facial nerve palsy, parotid gland enlargement, anterior uveitis, and low grade fever [1]. Heerfordt described three patients with uveitis, parotid swelling, cranial nerve palsy, and fever in 1909 [1] and Waldenström classified it as a distinct manifestation of sarcoidosis in 1937 [2]. A diagnosis of Heerfordt's syndrome can usually be made with confidence on the basis of characteristic clinical features. The simultaneous presence of all symptoms represents the complete form of this syndrome, with the complete form constituting 0.3% of all cases of sarcoidosis [3]. Because of its rarity, there are few case reports of Heerfordt's syndrome in the English literature [4–7]. Here, we report two cases of Heerfordt's syndrome.

## 2. Case Presentation

### 2.1. Case  1

A 53-year-old man presented with swelling of his right eyelid in March and was referred to the Department of Dermatology in a local hospital. A biopsy specimen from his eyelid revealed granulomatous blepharitis, and a granulomatous disease such as sarcoidosis was suspected. In spite of detailed examination, no uveitis or bilateral hilar lymphadenopathy (BHL) was detected and he was followed up closely without treatment. He was admitted to the Department of Dermatology in our hospital in August, because of worsening swelling of his right eyelid. As he also presented with a 3-month history of swelling of the right parotid gland and a 1-month history of right facial palsy, he was referred to the Department of Otolaryngology.

**Figure 1:**
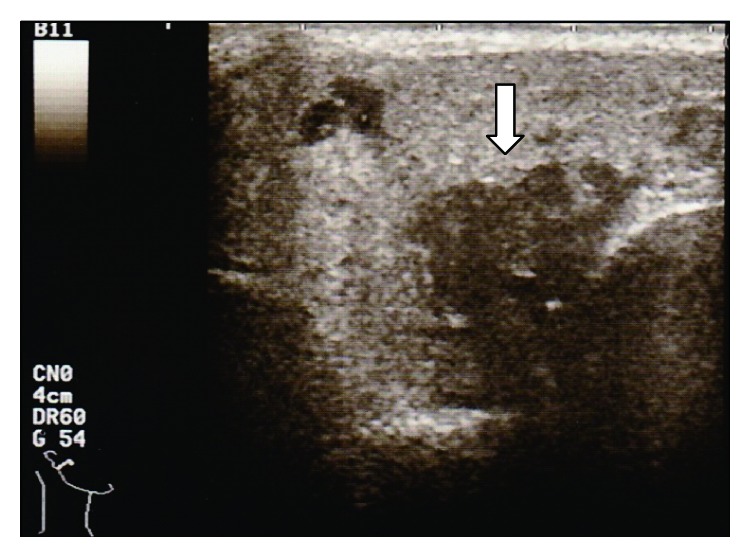
Ultrasound image for Case  1, showing the enlarged right parotid gland and interspersed hypoechoic areas (arrow).

**Figure 2:**
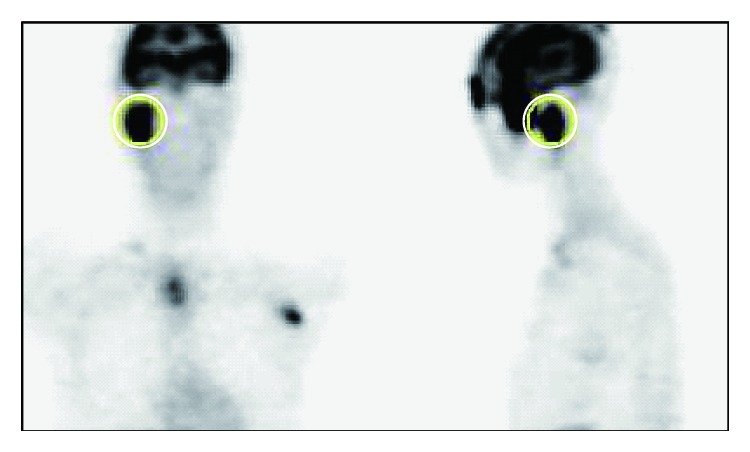
Positron emission tomographic image for Case  1, showing hypermetabolic activity in the right parotid gland (circles), right eyelid, and anterior mediastinal lymph nodes.

**Figure 3:**
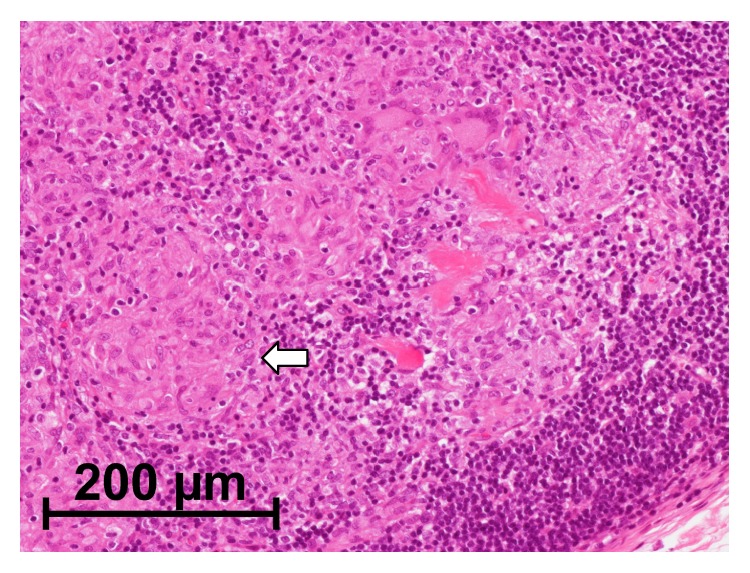
Histopathologic examination from the lymph node in the left thigh in Case  1, showing noncaseating epithelioid cell granuloma (arrow).

Physical examination revealed a swollen right eyelid and right parotid gland. He presented with a regular general status. Right facial nerve palsy especially in the forehead was also detected (House-Blackmann (HB) grade II). A pure tone audiogram showed normal hearing and stapedial reflex on the right side was normal. The patient did not complain of impaired taste. Ultrasound (US) examination showed an enlarged right parotid gland interspersed with hypoechoic areas (Figure 1). Electrophysiological tests, including electroneurography (ENoG) and the nerve excitability test (NET), showed no severe damage to the facial nerve, which indicated a good prognosis for the palsy. Positron emission tomography (PET) showed hypermetabolic activity in the right parotid gland, right eyelid, anterior mediastinal lymph nodes, groin lymph nodes, and subcutaneous nodule in the thigh (Figure 2). Serum angiotensin-converting enzyme (ACE) and soluble interleukin-2 receptor (sIL-2R) levels were elevated. Although uveitis was not detected in the ophthalmologic examination, biopsy specimens from the right eyelid and the lymph node in the left thigh revealed noncaseating epithelioid cell granuloma and a diagnosis of sarcoidosis was made histologically (Figure 3). Cardiac sarcoidosis was also suspected on the basis of electrocardiogram results showing premature ventricular contraction. Finally, we diagnosed this case as an incomplete form of Heerfordt's syndrome based on the absence of uveitis.

Oral corticosteroid therapy (prednisolone 40 mg per day) was started and the swelling of the right parotid gland diminished immediately. Asymmetry of the forehead continued for a few months, while the right facial nerve palsy gradually improved and was resolved completely at 4 months. Prednisolone was tapered carefully and stopped after 2 years. No recurrence has been observed to date.

### 2.2. Case  2

A 55-year-old woman visited our hospital due to left facial palsy and bilateral hearing loss lasting for 4 days. She had a history of postherpetic neuralgia and purpura pigmentosa chronica. As uveitis and BHL had been previously diagnosed, she underwent a detailed examination for sarcoidosis in the Department of Respiratory Medicine in our hospital.

**Figure 4:**
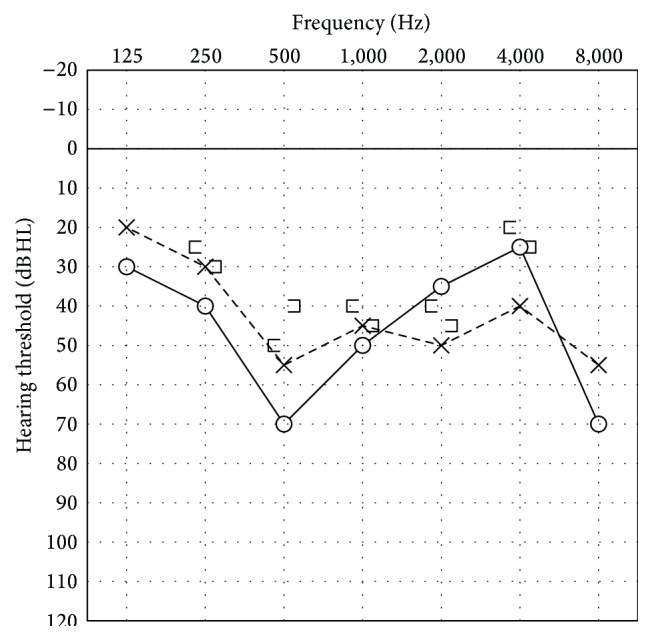
Pure tone audiogram for Case  2, showing moderate bilateral sensorineural hearing loss.

**Figure 5:**
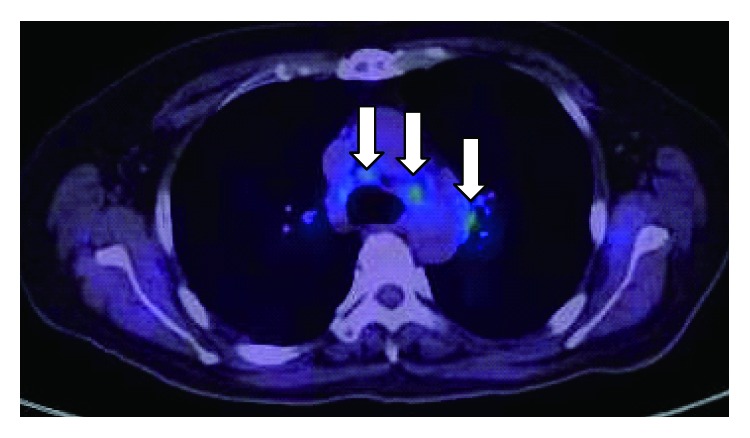
Positron emission tomographic image for Case  2, showing hypermetabolic activity in the bilateral hilar lymph nodes and mediastinal lymph nodes (arrows).

Physical examination revealed swelling of the bilateral parotid glands and her general condition was good. No nodules were detected and diffuse swelling of parotid gland was observed in the US examination. The facial palsy of left side was categorized as HB grade III and bilateral sensorineural hearing loss was detected by pure tone audiometry (Figure 4). Although geotropic direction-changing positional nystagmus was observed, no canal paresis was revealed by caloric test. The patient was negative bilaterally for stapedial reflex. NET showed no severe damage to the facial nerve. PET showed hypermetabolic activity in the bilateral hilar lymph nodes, mediastinal lymph nodes, lung, spleen, and myocardium (Figure 5). Magnetic resonance imaging with gadolinium enhancement revealed no lesions in her brain. Serum ACE and sIL-2R levels were elevated; however, there was no significant elevation in serum antibody for varicella zoster virus.

From these results, Heerfordt's syndrome was highly suspected; however, diagnosis was not confirmed histologically. As a result of consultation with the Department of Respiratory Medicine, treatment with prednisolone was started based on the premise that there was a possibility of a delayed histological confirmation of the diagnosis. She was treated with prednisolone at 60 mg per day, with the dose tapered after two weeks according to our therapeutic strategy for Bell's palsy. The swelling of the parotid glands improved immediately and the facial nerve palsy was resolved two weeks after the treatment. However, the patient required urgent hospitalization for heart failure after 2 months. Cardiac sarcoidosis was strongly suspected as the cause of the heart failure.

## 3. Discussion

Heerfordt's syndrome is characterized by the presence of facial nerve palsy, parotid gland enlargement, anterior uveitis, and low grade fever [1]. Nowadays, this syndrome is considered as a subtype of sarcoidosis, with the complete form of Heerfordt's syndrome constituting only 0.3% of all cases of sarcoidosis [3]. According to the diagnostic guidelines for sarcoidosis proposed by the Japan Society of Sarcoidosis and other Granulomatous Disorders in 2006 [8], Heerfordt's syndrome is classified into complete type, in which all four main symptoms are presented, and incomplete type, in which two out of the three symptoms of facial nerve palsy, parotid gland enlargement, and anterior uveitis are detected.

The standard treatment for Heerfordt's syndrome has not yet been established due to the rarity of this syndrome. However, treatment based on that for neurosarcoidosis should be indicated for Heerfordt's syndrome, in which facial nerve palsy is frequently observed. Neurological complications occur in 5–15% of sarcoidosis patients, and the facial nerve is one of the most frequently affected cranial nerves [9]. Corticosteroids are thought to be the first-choice treatment in the management of neurosarcoidosis in order to suppress inflammation, although prospective, double-blinded clinical trials have not yet been administered [9–11]. Although initial response rates to corticosteroids are high, a relapse of the symptoms may occur during the tapering of the corticosteroid dose. In these cases, immunosuppressant agents, including azathioprine, methotrexate, cyclosporine A, and cyclophosphamide, are used in combination with the corticosteroids [9, 10]. Cranial nerve palsy in neurosarcoidosis may be caused by nerve granulomas, perineural inflammatory infiltrates, increased cranial pressure, or granulomatous basal meningitis [11, 12]. Further, there is a possibility that epineurial necrotizing vasculitis could also lead to nerve ischemia with subsequent axonal degeneration [13]. There are some hypotheses regarding the site of the facial nerve lesion in Heerfordt's syndrome. Facial nerve palsy is thought to be the result of direct involvement of the facial nerve branches by the parotid lesion [14]. This is the most reasonable cause in cases in which nodular lesions are detected in the patients' parotid gland (i.e., Case  1). On the other hand, the facial nerve lesion might be in the internal auditory canal or intratemporal fallopian canal in the cases with hearing loss and/or vertigo (i.e., Case  2). Cases with loss of taste and hyperacusis have also been reported [7]. The presence of these cases implies the site of the facial nerve lesion is not limited to the parotid gland.

In Case  2, histological confirmation was not obtained at the onset of facial nerve palsy, although sarcoidosis was highly suspected based on the presence of uveitis, BHL, and the elevation in ACE. Treatment for the facial nerve palsy was given priority over the precise diagnosis of sarcoidosis after consultation with the Department of Respiratory Medicine. We used corticosteroids for two weeks based on our therapeutic strategy for Bell's palsy and the facial nerve palsy was observed to improve immediately. However, due to the delay in the histological confirmation of sarcoidosis, deterioration of the underlying cardiac sarcoidosis resulted in heart failure. As the prognosis for facial nerve palsy in sarcoidosis is thought to be good [14, 15], detailed examination, including bronchoscopic biopsy, should proceed in advance of the treatment for facial nerve palsy.

Sarcoidosis is a relatively uncommon disease for the otolaryngologist. However, the otolaryngologist may encounter Heerfordt's syndrome as this syndrome presents with facial nerve palsy and swelling of the parotid gland. Therefore, we should diagnose and treat Heerfordt's syndrome appropriately in cooperation with specialists in respiratory medicine and ophthalmologists.
